# Static and Dynamic Postural Changes after a Mountain Ultra-Marathon of 80 km and 5500 D+

**DOI:** 10.1371/journal.pone.0155085

**Published:** 2016-05-09

**Authors:** Giuseppe Marcolin, Alessandro Grainer, Carlo Reggiani, Patrizia Bisiacchi, Giorgia Cona, Nicola Petrone, Antonio Paoli

**Affiliations:** 1 Department of Biomedical Sciences, University of Padova, Padova, Italy; 2 Department of General Psychology, University of Padova, Padova, Italy; 3 Department of Industrial Engineering, University of Padova, Padova, Italy; University of Rome Foro Italico, ITALY

## Abstract

The study aimed to investigate the effect of fatigue on static and dynamic postural stability after completing a mountain ultra-marathon. Twelve male athletes participated in the study. Postural stability was assessed before and immediately after the race. Static postural stability was evaluated on a dynamometric platform with eyes opened (OE) and closed (CE). Dynamic postural stability was assessed with OE on an instrumented plate which allowed medio-lateral oscillations. Stabilometric data were affected by fatigue in the OE condition, concerning sway path velocity (p = 0.0006), sway area velocity (p = 0.0006), area of the confidence ellipse (p = 0.0016), maximal anterior-posterior (AP) (p = 0.0017) and medio-lateral (ML) (p = 0.0039) oscillations. In the CE condition the sway path velocity (p = 0.0334), the maximal ML oscillations (p = 0.0161) and the area of the confident ellipse (p = 0.0180) were also negatively influenced. Stabilogram diffusion analysis showed in the OE condition an increase of short-term diffusion coefficients considering the anterior-posterior direction (*Dfys;* p = 0.0023) and the combination of the two *(Dfr*^*2*^*s;* p = 0.0032). Equally, long term diffusion coefficients increased considering the anterior-posterior direction (*Dfyl;* p = 0.0093) and the combination of the two (*Dfr*^*2*^*l;* p = 0.0086). In CE condition greater values were detected for medio-lateral direction (*Dfxl;* p = 0.033), anterior-posterior direction (*Dfyl;* p = 0.0459) and the combination of the two (*Dfr*^*2*^*l;* p = 0.0048). The dynamic postural stability test showed an increase of the time spent with the edges of the plate on the floor (p = 0.0152). Our results showed that mountain ultra-marathon altered static stability more than dynamic stability. An involvement of cognitive resources to monitor postural stability after fatiguing could be the explanation of the worsening in the automatic task (quiet standing) and of the positive compensation in the less automatic task (dynamic standing on the instrumented plate).

## Introduction

The maintenance of postural stability depends on the complex interaction among visual, vestibular and somatosensory systems to keep the centre of gravity (CoG) within the base of support. To ensure this condition in an upright posture, contractions of several postural muscles controlled by the central nervous system are needed [1]. From a biomechanical point of view the measurement of the centre of pressure (CoP) displacement by means of force platforms is the most employed system to evaluate static upright posture. This methodology of postural assessment proved good intra-session and inter-session reliability, above all referred to sway length, CoP X and CoP Y parameters [2]. In addition to standard postural sway parameters, Collins and De Luca [3] introduced the stabilogram diffusion analysis (SDA) demonstrating that in a static upright posture, CoP trajectories could be modelled as a fractional Brownian motion and that two different neuro muscular control mechanisms operated: open-loop control schemes over short term intervals and closed loop control schemes over long-term intervals.

Postural control can be negatively influenced by fatigue protocols affecting specific muscles [4–8] as well as by prolonged exercises as cycling and running at different intensities [1,9,10]. Post exercise balance impairment is usually associated to prolonged exercise and recent findings also showed how in short intensive exercises the increase of postural sway is due to the hyperventilation more than to muscle fatigue [11].

In the last few years mountain ultra-marathons are experiencing an increase in popularity. They consist on running and walking uphill and downhill on different terrains for a longer distance than the classic marathon (42.195 km). Because of this extended length these competitions are a great opportunity to understand the effect of fatigue mechanisms when human body is pushed to its limit [12]. Moreover, the different kind of terrains together with the uphill and downhill running required a great postural stability control. Neuromuscular studies of mountain ultra-marathons showed a decrease in maximal voluntary contraction of knee extensors and plantar flexors, a modification of markers linked to muscle damage and inflammation as well as a failure of excitation-contraction coupling after the Ultra trail du Mont-Blanc (166 km length) [13]. Paradoxically a similar study investigating a more extreme race (330 km of length) underlined a less marked neuromuscular fatigue, muscle damage and inflammation, thus leading the authors to hypothesize a relative muscle preservation process [14]. An effect of muscle fatigue was also demonstrated for the respiratory muscles after a 110 km ultra-marathon with 5862 m of positive elevation gain [15].

Physiological consequences of an ultra-marathon are correlated with impairment of postural stability above all considering muscle fatigue due to the prolonged exercise at a sub-maximal intensity [11]. However to the best of our knowledge only one study in the literature investigated the effect of such an extreme race on the postural stability of the ultra runners by means of static tests on a stabilometric platform [16]. As expected authors find that standard posturographic parameters were significantly altered at the end of the race with an increase of the CoP length of 36% in the anterior posterior direction and 29% in the medio-lateral direction. Moreover stabilogram diffusion analysis (SDA) confirmed the effect of fatigue in both short term intervals and long term intervals appearing to be more sensitive than standard CoP parameters.

Starting from the results of Degache and colleagues [16] and considering that during an ultra mountain-marathon there is a great involvement of dynamic postural stability due to the steep slopes and to the different kind of terrains, the aim of the present work was to investigate the effect of an 80 km mountain ultra-marathon on static and dynamic postural stability. We hypothesized a more marked worsening of the dynamic postural stability with respect to the static postural stability.

## Methods

### Participants

Twelve male ultra-marathon runners who completed at least one mountain ultra-marathon the year before, were involved in the study (Age 46.5 ± 8.3 yr; weight 74.6 ± 7.9 kg; height 1.81 ± 0.8 m). One week before the race each participant received a written detailed description of the research protocol with the explanation of the postural stability tests. Athletes involved in the study read and signed an informed consent the day of the race. All testing procedures of the study were approved by the ethical committee of the Department of Biomedical Sciences, University of Padova (HEC 2–2013).

### Instruments and experimental design

Data were collected the 26^th^ and 27^th^ of July 2013, in occasion of the second edition of the Trans d’Havet race. This competition took place in the north-east of Italy and in the 2013 represented the Ultra race of the European Skyrunning® championships. The race track consisted of 80 km with a total elevation of 5500 m and a maximum altitude of 2238 m. The race started at 1 am and the maximum time allowed was 36 hours. The organizations guaranteed medical stations and rest stops with food and drinks along the whole race. Recovery time for nutrition and hydration was managed by each participant itself and was not subtracted from the final race time. However, each participant had to pass pre-defined gates not exceeding a fixed time in order to continue the race.

Postural stability of each athlete was tested twice: within 2 hours before starting the race (PRE condition) and 12 ± 3 minutes after crossing the finish line (POST condition). Each session consisted of a battery of two static postural stability tests, the first with opened eyes and the second with closed eyes, followed by one dynamic postural stability test. A dynamometric platform (RGMD S.p.a., Genova, Italy) was used for the static stability test to measure the trajectory of the CoP in the anterior-posterior (AP) and medio-lateral (ML) direction at a sampling rate of 100 Hz. The athletes had to stand on the platform without shoes, positioning their feet on specific lines drawn on the surface of the plate according to the manufacturer. In particular they had to form an angle of 30° between feet maintaining heels at the same constant distance. After that, athletes were instructed to assume an upright posture with extended legs and arms naturally positioned along their sides. In the first trial with opened eyes (OE) they were asked to look ahead fixing a target on the wall at 1 m of distance while in the second trial with closed eyes (CE) they were instructed to maintain the upright posture for all the duration of the trial keeping their eyes closed. Each participant performed one test of 30 seconds for each of the two conditions. The duration of the test was chosen accordingly to Scoppa and colleagues [17]. Rest between the trials was set to 60 seconds.

**Fig 1:**
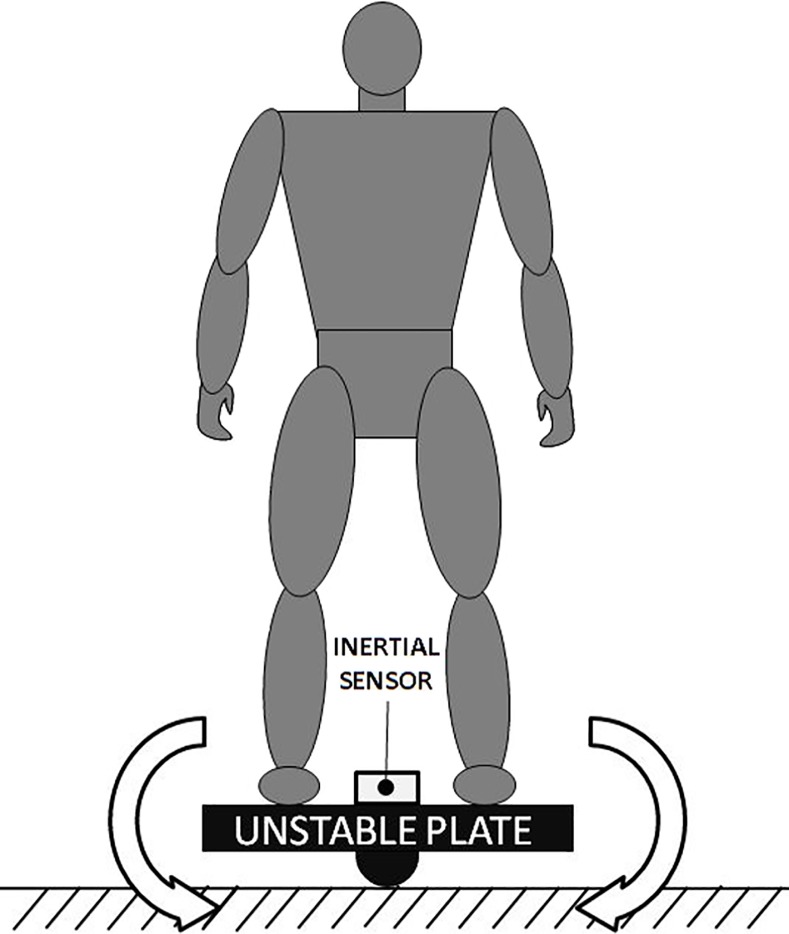
Schematic illustration of the experimental set up for the dynamic stability test on the unstable plate.

An unstable plate rotating only along a single axis and a MTw™ 3D inertial sensor with MEMS technology (Xsens Technologies B.V., Enschede, The Netherlands) were used in the dynamic stability test. MTw™ is a small and completely wireless 3D motion tracker sensor (dimensions 34.5 mm x 57.8 mm x 14.5 mm; weight 27 g) with a 3D linear accelerometer, a 3D gyroscope, a 3D magnetometer and barometer embedded in it. The sensor collects 3D orientation, 3D acceleration, 3D angular velocity, static pressure and earth-magnetic field intensity. The MTw™ sensor was positioned over the unstable plate with its x axis parallel to the rotational axis of the plate. The athlete was asked to stand on the plate with his sagittal axis parallel to the rotational axis of the unstable plate and the feet parallels, according to specific lines drawn on the surface of the plate. Subsequently the athlete was instructed to maintain the plate parallel to the floor as much as possible without moving the feet from their original position for the whole test (Fig 1). Arms and trunk movements for counter balance actions were allowed. The 3D inertial sensor measured the roll angle around its x axis that corresponded to the right and left oscillation of the subject around the rotational axis of the plate. Each subject performed one trial of 25 seconds with opened eyes (OE) accordingly with the indications of Scoppa and colleagues for stability tests [17]. The 3D inertial sensor sampling rate was set at 100Hz.

## Data Analysis

Maximal oscillation in the anterior-posterior (AP) and medio-lateral (ML) direction [mm], the sway path velocity [mm/sec], the area of the confidence ellipse [mm^2^] and the sway area velocity [mm^2^/sec] were calculated from CoP data recorded by the force platform. Further, sway area was calculated as the sum of the areas of the triangles having as vertexes the barycentre of the CoP path and two consecutive points of the CoP path; sway area velocity represented the area swept per time unit. Romberg coefficient was computed for all the variables described above. The analysis was performed for each athlete before and after the race, extracting data from each 30 seconds trial, both with OE and CE.

The stabilogram diffusion analysis (SDA) was referred to the diffusion coefficients (*Df*) because they reflect the level of stochastic activity of the CoP, thus representing an index of postural instability along the x (*Dfx*) and y (*Dfy*) axis or the combination of the two (*Dfr*^*2*^) considering the plane of support [3]. The square of the displacements between all pairs of point separated by a specific time interval ∆t was calculated for AP, ML, and the plane of support. Then they were averaged over the number of ∆t. This procedure was repeated for incremental values of ∆t that, according to the sample frequency of 100 Hz, ranged from 0.01 to 10 seconds with step of 0.01 seconds. The mean square displacements plotted versus the ∆t time interval represented the stabilogram diffusion plot. Two linear regression were fitted: one for the short-time period (time interval from 0 to 0.5) and one for the long-time period (time interval from 2 to 10). Diffusion coefficients were calculated as half the slopes of the two regressions. The analysis tool for SDA was developed in MATLAB (The MathWorks, Inc., MA, USA).

In the dynamic stability test, the 3D inertial sensor positioned over the unstable plate allowed to measure the rotation of the plate: when the sensor was parallel to the floor it measured 0°, when it rotated clockwise it measured positive values and when it rotated counter clockwise it measured negative values. Four parameters were determined to quantify the results of the dynamic stability test: the integral of the curve (Full Balance, FB), the time each athlete was able to stay between +5° and -5° (Fine Balance, FiB) and between +10° and -10° (Gross Balance, GB) and the time the plate stayed with its right and left edge on the floor (Stay Time, ST). Last, the time the edge of the plate stayed on the floor was subtracted from the 25 seconds of the trial and again it was calculated the percentage the subject maintained the plate between +5° and -5° (Net Fine Balance, NFiB%) and between +10° and -10° (Net Gross Balance, NGB%).

### Statistical analysis

For both static and dynamic postural stability tests the two-tailed paired t-test was used to compare each dependent variable before and after the mountain ultra-marathon. Kolmogorov-Smirnov (KS) test was employed to check normality distribution. The level of significance was set for p ≤ 0.05. Data analysis was performed using the software package GraphPad Prism 4.00 (GraphPad Software, San Diego California USA). Statistical Power and Effect size were both calculated with the G*Power 3.1.5 software [18].

## Results

Athletes involved in the study finished the race with an average time of 15 hours and 56 minutes ± 2 hours and 3 minutes and they ranged in the official ranking from the 35^th^ to the 204^th^ place. All data reported below are presented as mean and standard deviation.

### Static stability tests

The OE condition showed statistically significant differences between the PRE and POST condition concerning sway path velocity (p = 0.0006; Effect Size: 1.19; Power: 0.36), sway area velocity (p = 0.0006; Effect Size: 1.15; Power: 0.32), area of the confidence ellipse (p = 0.0016; Effect Size: 1.27; Power: 0.61), maximal AP oscillations (p = 0.0017; Effect Size: 1.32; Power: 0.66), maximal ML oscillations (p = 0.0039; Effect Size: 1.24; Power: 0.72).

In the CE condition the race significantly influenced the sway path velocity (p = 0.0334; Effect Size: 0.51; Power: 0.29), the maximal ML oscillations (p = 0.0161; Effect Size: 0.91; Power: 0.62) and the area of the confident ellipse (p = 0.0180; Effect Size: 0.83; Power: 0.56). Results relative to the static postural test are summarized in Table 1 and Table 2.

**Table 1:** Results referred to the static test on the stabilometric platform.

	Opened eyes (OE)			Closed eyes (CE)		
	PRE	POST	*p*	PRE	POST	*p*
Sway path velocity [mm/sec]	9.19 ± 2.78	13.81 ± 4.44	*	13.25 ± 5.30	17.26 ± 9.06	§
Sway area velocity [mm^2^/sec]	13.13 ± 7.83	27.36 ± 14.30	*	18.42 ± 8.75	32.76 ± 29.07	
Confident ellipse area [mm^2^]	120.6 ± 57.38	287.4 ± 150.1	*	138.4 ± 63.47	240.7 ± 141.6	§
AP oscillations[mm]	19.39 ± 4.86	31.64 ± 10.74	*	22.20 ± 8.14	25.76 ± 8.07	
ML oscillations[mm]	12.62 ± 2.32	19.13 ± 6	*	13.80 ± 5.52	20.61 ± 8.56	§

* p<0.01.

§ p<0.05.

**Table 2:** Results of Romberg coefficient relative to the static stability tests on the stabilometric platform.

	Romberg Coefficient	
	PRE	POST
Sway path velocity [mm/sec]	1.42 ± 0.30	1.22 ± 0.34
Sway area velocity [mm^2^/sec]	1.52 ± 0.61	1.10 ± 0.37
Confident ellipse area [mm^2^]	1.28 ± 0.57	0.86 ± 0.23
AP oscillations [mm]	1.17 ± 0.38	0.85 ± 0.27
ML oscillations [mm]	1.09 ± 0.37	1.09 ± 0.31

**Fig 2:**
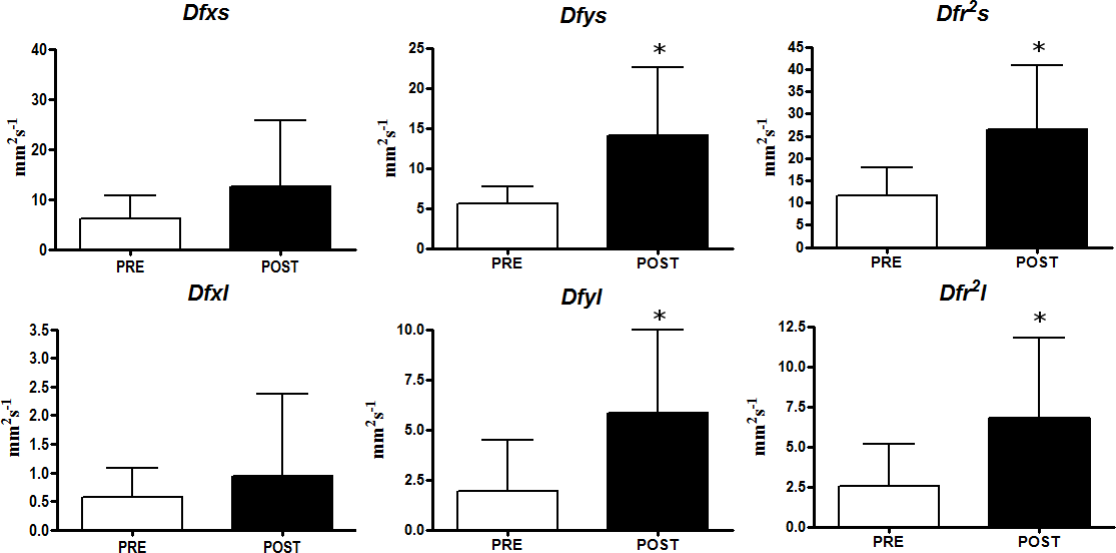
Stabilogram Diffusion Analysis results with opened eyes (OE). (* p<0.01; § p<0.05).

**Fig 3:**
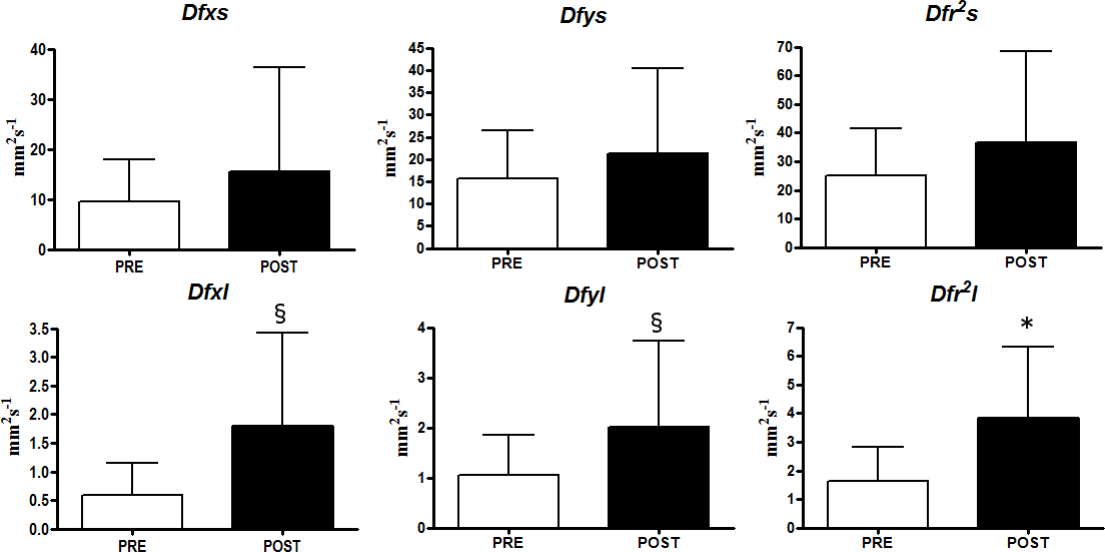
Stabilogram Diffusion Analysis results with closed eyes (CE). (* p<0.01; § p<0.05).

SDA analysis allowed to appreciate further differences comparing the PRE race to the POST race conditions. The short-term *Dfr*^*2*^*s* and the long term *Dfr*^*2*^*l* with OE (Fig 2) significantly increased in the POST race with respect to the PRE race (p = 0.0032; Effect Size: 1.19; Power: 0.64 and p = 0.0086; Effect Size: 0.99; Power: 0.61, respectively). Moreover statistically significant increase was detected for the short term *Dfys* (p = 0.0023; Effect Size: 1.11; Power: 0.50) and the long term *Dfyl* (p = 0.0093; Effect Size: 1.08; Power: 0.71). With CE (Fig 3) differences were detected between PRE and POST race for *Dfr*^*2*^*l* (p = 0.0048; Effect Size: 1.00; Power: 0.52), *Dfxl* (p = 0.033; Effect Size: 0.86; Power: 0.70), *Dfyl* (p = 0.0459; Effect Size: 0.65; Power: 0.52).

### Dynamic stability test

Results relative to the dynamic stability test are presented in Table 3. Significant differences were reported only in stay time (ST) with an increase in the POST condition with respect to the PRE condition (p = 0.0152; Effect Size: 0.88; Power: 0.78).

**Table 3:** Results referred to the dynamic test on the instrumented platform.

	Opened eyes (OE)		
	PRE	POST	p
**FB**-Full Balance	258.0 ± 21.82	258.4 ± 26.05	
**FiB**-Fine Balance [sec]	5.29 ± 1.36	5.89 ± 2.06	
**GB**-Gross Balance [sec]	10.74 ± 1.93	10.43 ± 2.18	
**ST**-Stay Time [sec]	6.2 ± 1.29	7.64 ± 1.85	§
**NFiB**-Net Fine Balance [%]	28.08 ± 6.33	33.8 ± 10.86	
**NGB**-Net Gross Balance [%]	57.05 ± 8.65	60.07 ± 10.56	

§ p<0.05.

## Discussion

Muscular exercise is recognised to be one of the causes of postural stability deterioration in human subjects and several factors contribute to its worsening. Further, body sway may be influenced by the intensity of the exercise [1,9,19,20] and by the type of exercise [6,11,21]. In the present study we focused our attention in postural stability variations after an 80 km mountain ultra-marathon by means of a combination of static and dynamic stability tests. Considering the muscle effort and the duration of the race, this competition could represent a good model to investigate the consequences the alterations of neuromuscular functions had on the postural stability control hypothesizing a more marked worsening of the dynamic postural stability with respect to the static postural stability.

The increase of CoP standard posturographic parameters detected are in accord with previous investigations considering general muscular exercise [10,16,20,21] and local muscular exercise [4,7,8,22]. These alterations in the postural stability control can be related to the muscular effort but also to an impairment of the system’s capacity to control the position of the centre of gravity [20]. However a more marked worsening was detected in the OE condition where all the parameters showed a statistically significant increase. On the other hand the CE condition revealed higher values only for sway path velocity, area of the confident ellipse and ML maximal oscillations.

This less pronounced worsening of the classic stabilometric parameters with CE condition in the POST race could seem controversial if we consider how the visual input usually compensate the alteration of the others regulatory systems [1,23]. We referred in particular to the compensation of the proprioceptive system, that should be certainly less efficient after 80 km of uphill and downhill. However, it has to be acknowledged that there is a continuous stimulation of visual input by the moving field of vision with the evocation of movement in the opposite direction to the perceived one during running [6]: postural imbalances observed in OE condition after the exercise could be the results of this continuous stimulation [23]. Therefore, the more marked worsening detected in the OE condition after the race can be explained by the aforementioned stimulation of the visual input that could have reduced its compensative contribute to the balance control.

The analysis of stabilograms as fractional Brownian motion allowed to individualize two distinct neuromuscular control mechanisms during quiet standing: open loop control schemes employed over short term intervals and closed loop control mechanisms, employed over long term intervals [3]. In particular Diffusion coefficients represent the level of stochastic activity of the CoP considering the medio-lateral (*Dfx*) and the antero-posterior (*Dfy*) axis as well as the plane of support (*Dfr*^*2*^). Higher values of Diffusion coefficients indicate a less tightly regulated or more random control system [3]. To this extent, the results of our study are similar to those reported by Degache et al. [16] showing an overall increase of Diffusion coefficients immediately after the end of the race. In the OE condition, the increase of *Dfr*^*2*^*s* and *Dfr*^*2*^*l* reflected the results of the classical stabilometric parameters. However, considering the two single axes, only Diffusion coefficients referred to the anterior posterior one (*Dfys* and *Dfyl)* showed statistically significant increments. This result was in part expected considering that athletes involved in mountain ultra-marathons reported great level of fatigue of plantar flexors [13,14] that could negatively influences the control movement along this axis [16]. In the CE condition, the stochastic activity of the CoP significantly increased only considering the long term intervals (*Dfr*^*2*^*l*, *Dfxl*, *Dfyl*) that is when corrective feedback mechanisms are activated. These results relative to classical stabilometric parameters and to stabilogram diffusion analysis both supported the thesis of Derave et al. [23]: exercise of moderate intensity deteriorate visual contribution in maintaining quiet standing posture. Therefore we can speculate that, after a mountain ultra-marathon race, the disturbance of the visual information seems to be more relevant in comparison with the disturbance of the proprioceptive information in the quiet standing postural stability control.

Results relative to the dynamic postural stability test on the instrumented plate showed a statistically significant increment of ST that was the time spent with one of the two edges of the plate touching the floor. It has been demonstrated how fatigue could have a negative effect not only in force producing capacity during side-step cutting task, but also in performing a smooth and controlled action [24]. We can speculate that after a mountain ultra-marathon, fatigue does not allow to perform a continuous and controlled dynamic movement on the unstable plate, thus introducing a series of static breaks on the edges within the dynamic postural stability test itself. Surprisingly, no statistically significant changes were detected in the medio-lateral oscillation during the dynamic postural stability test. However these results are supported by the fact that in the static postural stability test with OE, also *Dfxs* and *Dfxl* did not show statistically significant changes and so no variations in the level of stochastic activity of the CoP relative to medio-lateral oscillations. Therefore our data support the thesis of a more marked fatigue of plantar-flexors muscles that are involved in the anterior-posterior postural stability control, with respect to abductor and adductor muscles, employed in the medio-lateral postural stability control.

The comparison of static and dynamic results of this study seems to be conflicting showing an overall decrease of postural stability in the dynamometric platform while no worsening was detected relative to the dynamic stability test on the unstable plate except for the ST parameter. First, it has to be considered that maintaining quiet standing is linked to automatic control processes while trying to stay in an unstable plate required a marked voluntary control process. Further, on one side it is known how attentional focus on body sway promoted the use of less automatic control process with a less efficient posture control during quiet standing [25]. On the other side the exercise induced fatigue can be mitigated by a cognitive compensation [6]. Taking together these considerations we can speculate that the athletes of the present study increased their cognitive resources in the tests after the 80 km competition to contrast the effect of fatigue. This behaviour could have lead to an overall worsening in the more automatic task (the quiet standing) and a compensation in the less automatic task (the dynamic stability on the instrumented plate).

In conclusion our result showed that mountain ultra-marathon altered more static than dynamic stability. In this kind of competitions dynamic stability is certainly more important than static stability because of the unstable terrain and the uphill/downhill running. The increasing of cognitive resources seemed to be the choice adopted by athletes to compensate the negative effect of muscle fatigue, thus reducing the worsening of dynamic stability. Consequently this strategy together with the alteration of the visual contribution could have been the cause of the more marked worsening in the static stability tests. However our conclusions need to be confirmed with further researches. Moreover, our findings induced to consider that further studies should focus in the assessment of dynamic balance rather than static balance with the ultimate goal of finding a potential correlation between specific parameters and injuries in mountain ultra-marathons.

Finally some limitations of the study have to be acknowledged. First, a larger sample size could have improved statistical power but it is difficult in such extreme races to enrol a large number of athletes asking them to come to the starting point several hours before the beginning of the competition. Moreover it is very difficult to complete such a strenuous competition where lots of athletes withdrew because of several hindrances as muscle injuries, trauma, gastrointestinal troubles or, from a psychological point of view, loss of motivation.
